# Supplementary figures and images for: Evolution of innate behavioral strategies through competitive population dynamics
Source: PLoS Comput Biol. 2022 Mar 14;18(3):e1009934. doi: 10.1371/journal.pcbi.1009934 (PMC8947601; doi:10.1371/journal.pcbi.1009934)

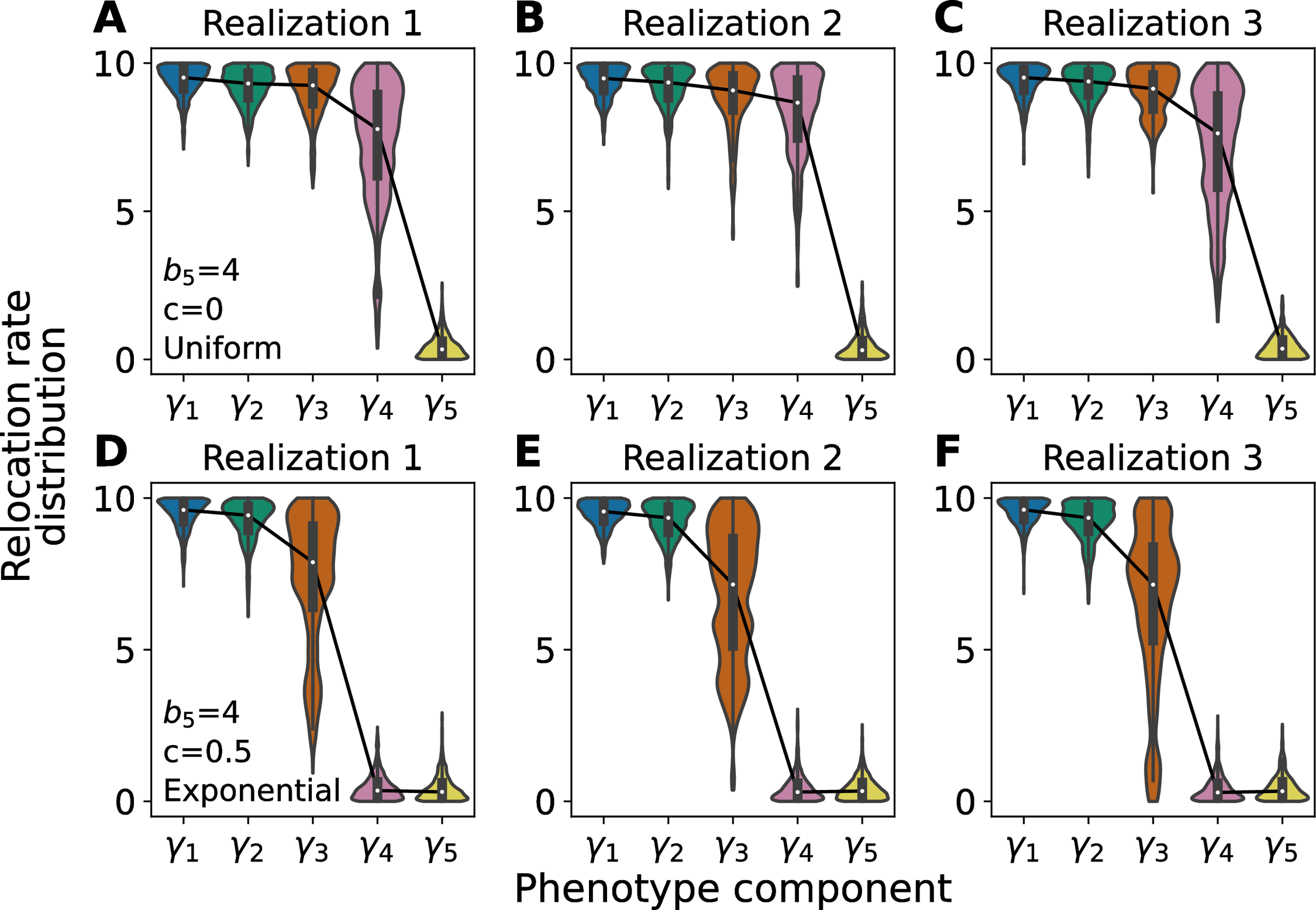

Supplement: S1 Fig — In evaluating the phenotype distributions shown in the main text, we average over 20 trials of the evolutionary dynamics, but we keep the random realization of the resource distribution the same across these 20 trials. To demonstrate that the specific realization of resources throughout the environment does not strongly shape the ultimate phenotype distribution, we run the agent-based stochastic simulations for additional unique random realizations of the resources throughout the environment. A-C. Phenotype distribution at 20000 iterations, for 3 different realizations of resource levels drawn from a discrete uniform distribution. D-F. Phenotype distributions at 20000 iterations, for 3 different realizations drawn from an exponential distribution wk ∝ e−ck with c = 0.5. In both cases, different realizations of the resource give us consistent phenotype distributions. (TIF) [file pcbi.1009934.s001.tif]

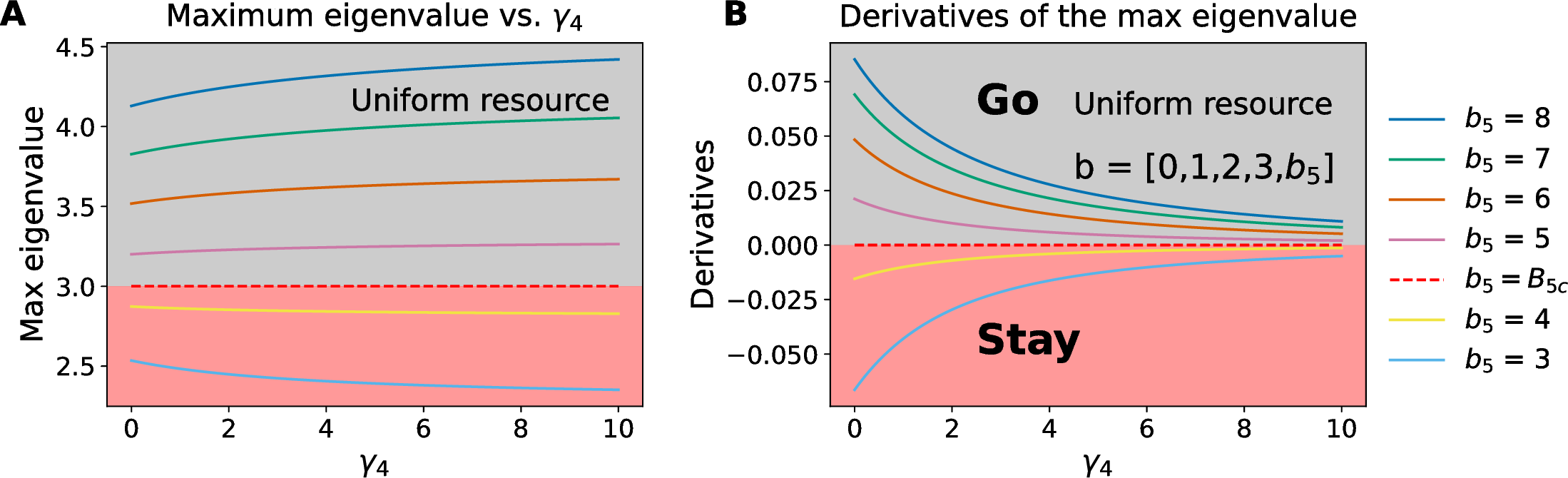

Supplement: S2 Fig — As discussed in the main text, the maximum eigenvalue behaves as an effective objective function and predicts the transition of γ4 when b5 is varied. It also explains the large variability of the γ4 component, as the maximum eigenvalue is insensitive to γ4 when b5 ≈ B5c. A. The maximum eigenvalue Λγ of the low-density coefficient matrix Mγ predicts the most competitive foraging strategies γopt. Typically, the optimal values are γ1,2,3 = 10 and γ5 = 0, but the value of γ4 depends on the birth rate at resource level S5, b5. When b5 is above the critical value B5c, the phenotype with γ4 → γmax has the largest maximum eigenvalue and thus are predicted to be the optimal; however when b5 is below B5c, the phenotype with γ4 → γmin is predicted to dominate the population instead. The same transition in the full agent-based stochastic simulation is shown in Fig 3. B. The derivatives of the eigenvalue predict a range of γ4 values yield similar maximum eigenvalues when b5 is close to B5c. The effective objective function is insensitive to changes of γ4, resulting in a large variability of γ4 as seen in the simulation results shown in Fig 2E and 2F. When the derivative is positive, larger γ4 leads to larger λγ and thus agents are better off leaving their current location to explore (“go”). When the derivative is negative, smaller γ4 leads to larger λγ and thus agents are better off exploiting their current location (“stay”). (TIF) [file pcbi.1009934.s002.tif]

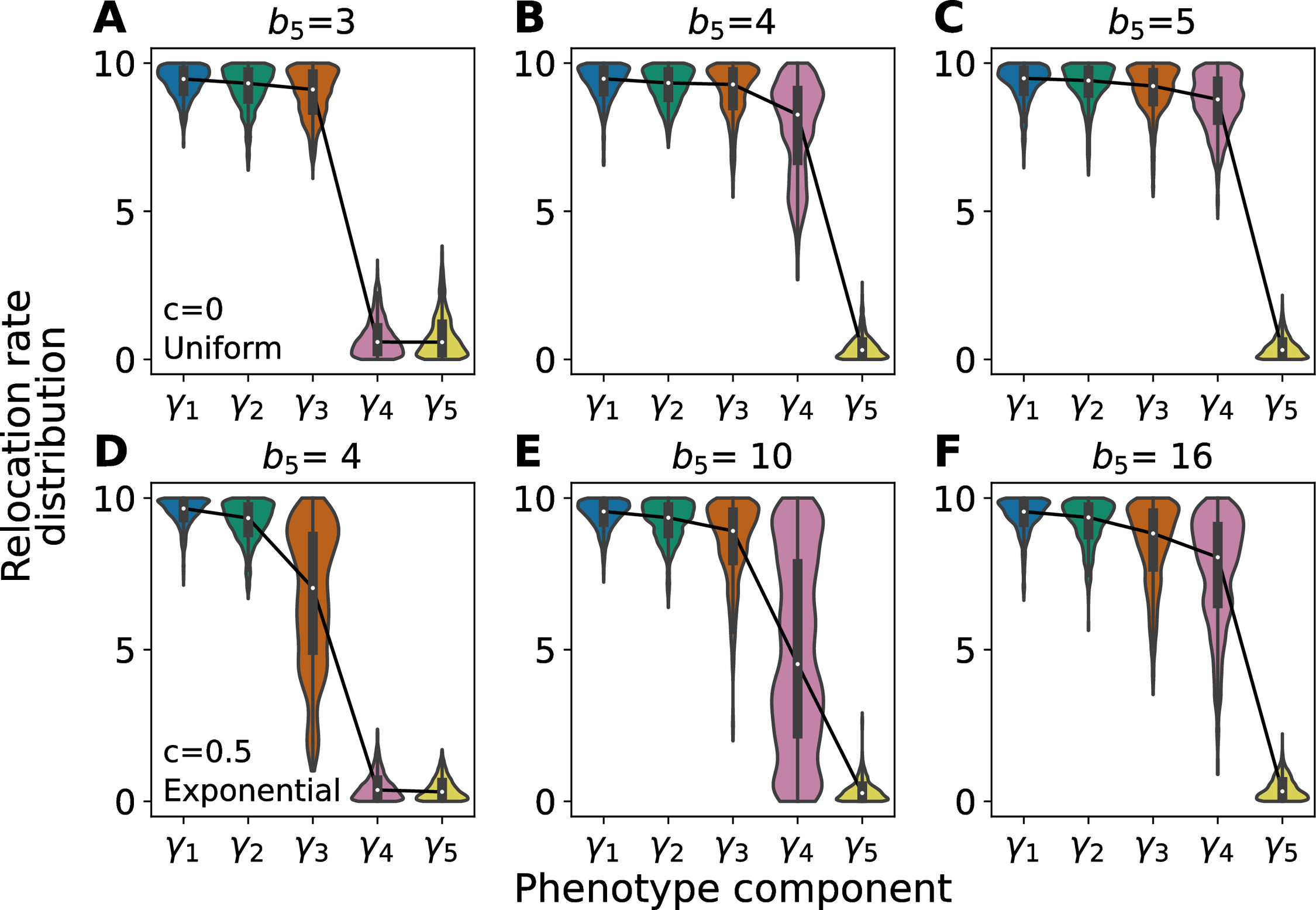

Supplement: S3 Fig — Changing the birth rate at the highest resource level b5 leads to a transition in the most competitive phenotypes, primarily in the fourth component γ4, as shown in Fig 3B. Here we show the full phenotype distributions for some representative cases around and at this transition, for both discrete uniform and exponentially distributed resource levels. A-C. Phenotype distribution at 20000 iterations for a discrete uniform resource distribution, for b5 of 4, 5, and 6, respectively. D-F. Phenotype distribution at 20000 iterations for an exponential resource distribution c = 0.5, for b5 of 4, 10, and 16, respectively. In all the cases, b1 = 0, b2 = 1, b3 = 2, b4 = 3. (TIF) [file pcbi.1009934.s003.tif]

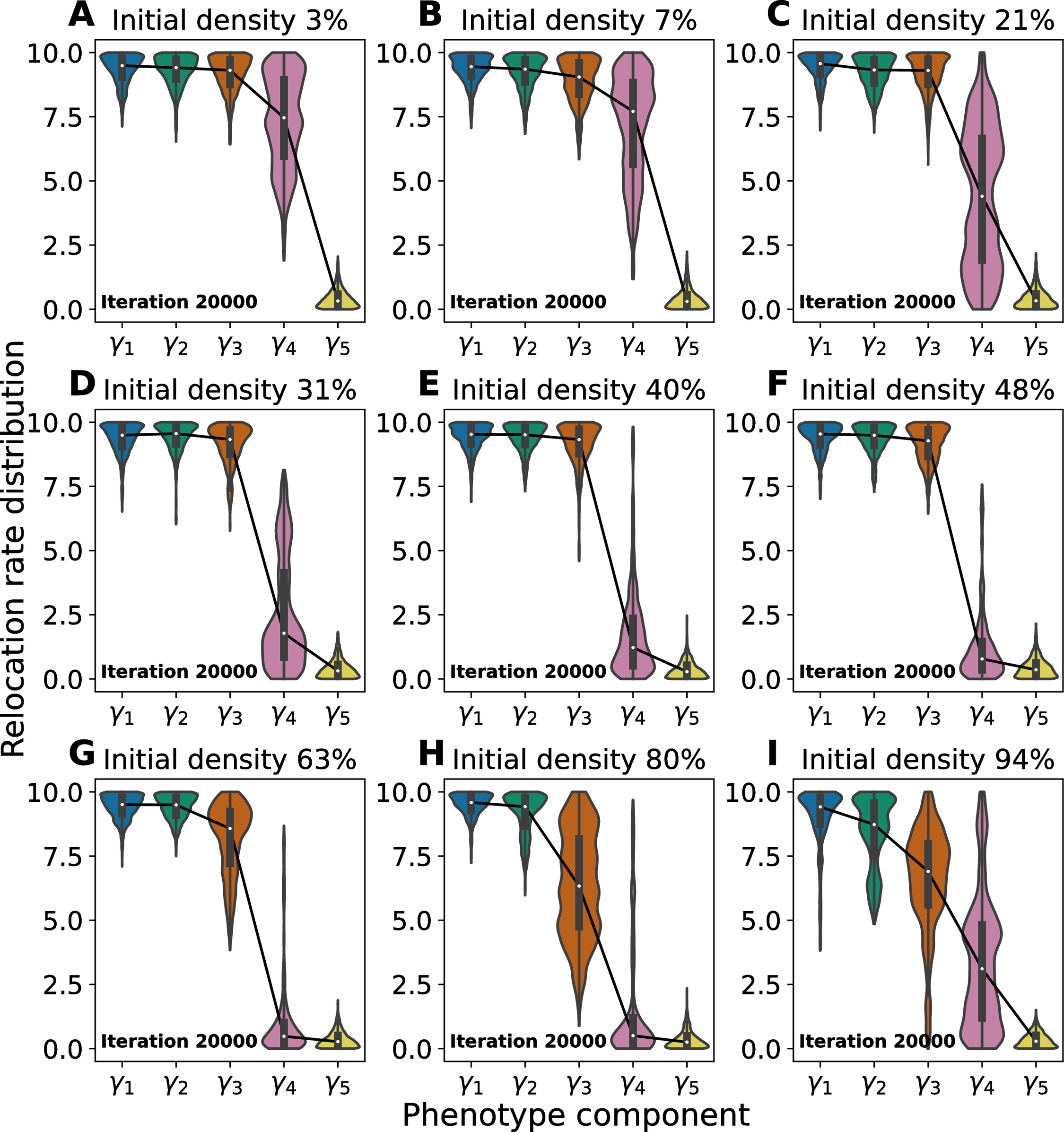

Supplement: S4 Fig — In the full agent-based stochastic simulation we observed that there exists a range of initial densities with which different phenotypes coexist for an evolutionarily long time, at least up to 20000 iterations of the full stochastic simulations shown in Fig 4. Here we present the violin plots of the relocation rate distributions of the full agent-based stochastic simulations at these different initial densities, showing the coexistence of phenotypes at a range of initial densities, indicated by a large width of the distribution. At lower densities, coexistence occurs when b5 ≈ B5c, while some degree of coexistence is observed for several initial densities without tuning b5. Note that the large degree of coexistence at 94% density is mostly due to the environment being so dense that opportunities for relocation and birth are extremely rare. (TIF) [file pcbi.1009934.s004.tif]

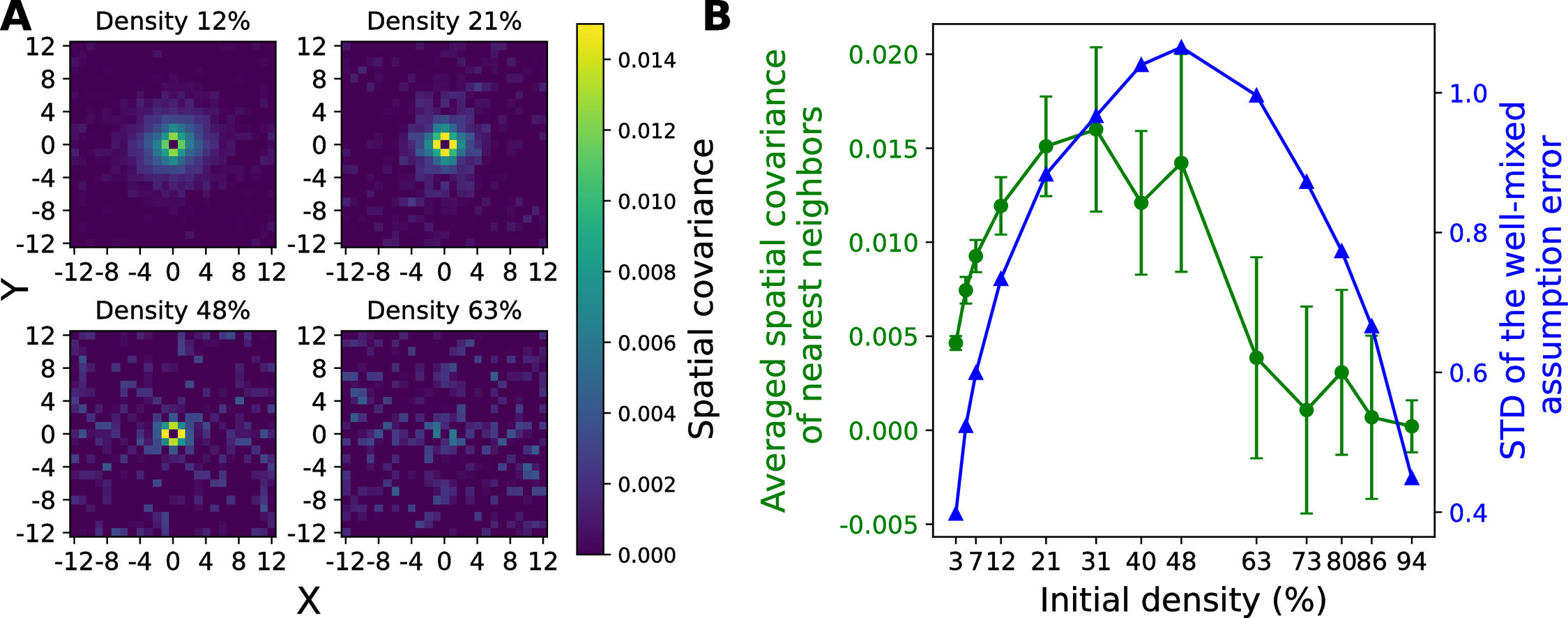

Supplement: S5 Fig — In deriving the 5-state mean-field model from the 2d master equation model, we made a “well-mixed approximation” (Eq (6)). The error introduced by this assumption (the difference of left and right hand sides of Eq (6)) has a zero mean when averaged across sites and here we show how the standard deviation of this error changes over different initial densities. To assess the spatial variation neglected by our 5-state approximation, we evaluated the spatial cross-covariance of the agent numbers across different sites and then averaged across trials. This cross-covariance is localized around the nearest neighbor sites at low-to-medium densities, but weakens at higher densities. Averaging the covariance of the 4 nearest neighbors exhibits qualitatively similar behavior as the standard deviation of the well-mixed assumption error as the initial density changes. A. The spatial covariance of the number of agents at location (x, y) with the number of agents at sites (x + i, y + j), averaged across all sites with periodic boundary conditions and then averaged over 20 trials of independent simulations. The center of the covariance map (the variance) is suppressed for visibility. B. Green line and circle markers show the averaged spatial covariance of the 4 nearest neighbors in panel A (i.e., the mean of the covariances at (1, 0), (-1, 0), (0, 1), and (0, -1)) at different initial density levels. Blue line and triangle markers show the standard deviation of the well-mixed assumption error (the difference of left and right hand sides of Eq (6)) across 20 trials at different initial density levels. Both quantities are small for very low or high densities, with peaks in a density range around 31% to 48%. (TIF) [file pcbi.1009934.s005.tif]

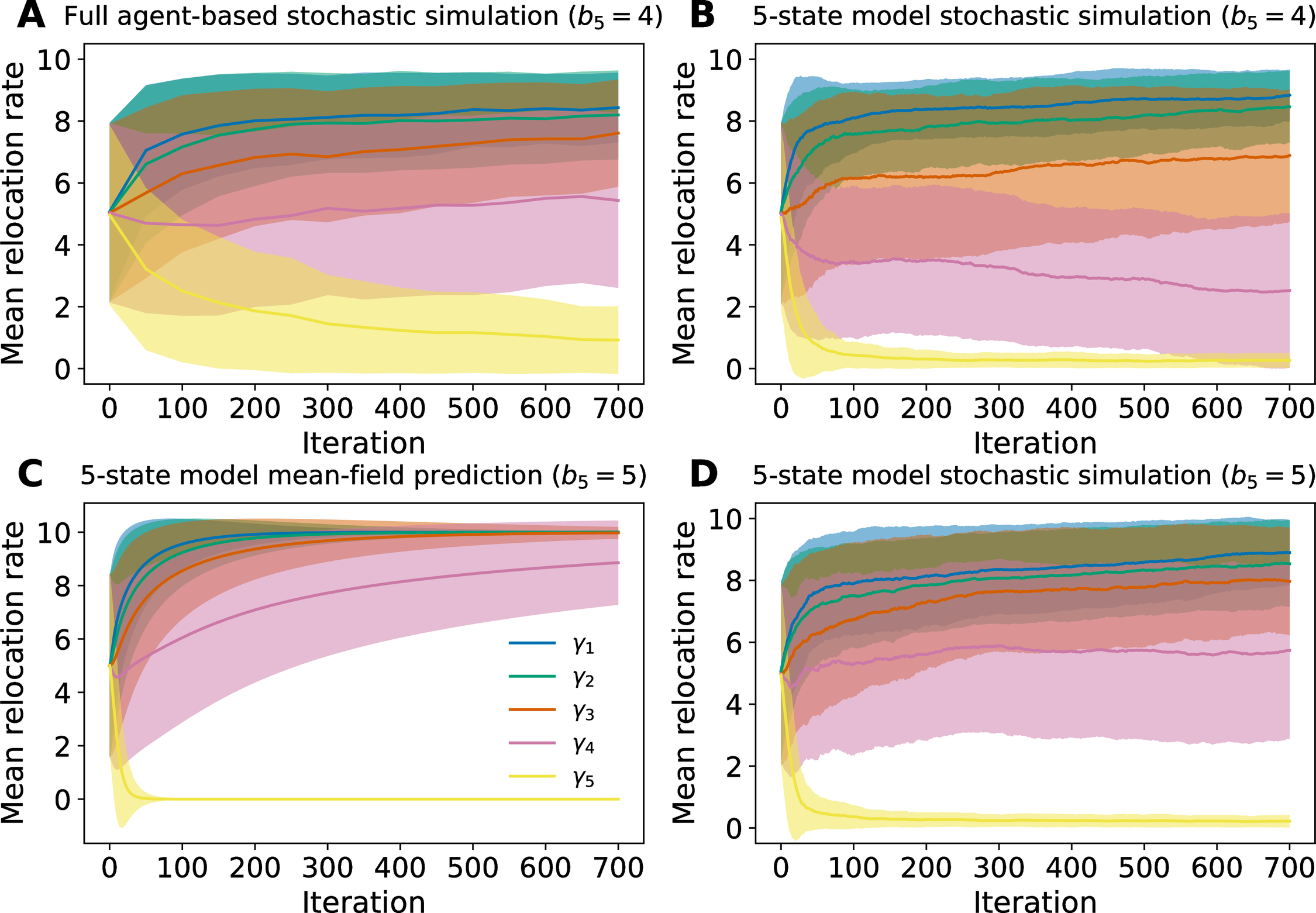

Supplement: S6 Fig — The full agent-based model has a population of agents navigating on a 2d environment. For analytical tractability, this full model is reduced to a 5-state model that eliminates spatial dependencies. However, it is observed in Fig 2E and 2F that the full model and the reduced model have a qualitative mismatch in the order of magnitude of the convergence speed, as well as a quantitative mismatch in the value of the transition point of b5. Here we show the comparison of the two models at a shorter timescale (700 iterations) with different b5 values (4 and 5). The results suggest that the well-mixed assumption introduces the discrepancy in timescales, as simulations of a stochastic 5-state model agree with the mean-field 5-state model. A. The mean relocation rates of the full 2d stochastic simulation with b5 = 4 have not converged by 700 catastrophes. B. Agent-based simulations of a 5-state model with b5 = 4 show a faster convergence of γ5 to 0 and γ1,2,3 to high values near γmax = 10, within the same 700 catastrophes shown in A (note the different scales of the horizontal axes in Fig 2E and 2F). C. The 5-state mean-field model with b5 = 5 (which predicts γ4 → 10 at long times) converges within 700 catastrophes, though the fate of γ4 is different from the stochastic 5-state model with b5 = 4. D. The stochastic agent-based 5-state model with b5 = 5 largely converges within 700 iterations and agrees slightly better with the mean-field model’s prediction for γ4. (TIF) [file pcbi.1009934.s006.tif]
